# Exosomal miR-200c-3p negatively regulates the migraion and invasion of lipopolysaccharide (LPS)-stimulated colorectal cancer (CRC)

**DOI:** 10.1186/s12860-020-00291-0

**Published:** 2020-06-29

**Authors:** Yimei Jiang, Xiaopin Ji, Kun Liu, Yiqing Shi, Changgang Wang, You Li, Tao Zhang, Yonggang He, Ming Xiang, Ren Zhao

**Affiliations:** 1 Department of General surgery, Ruijin Hospital North, Shanghai Jiaotong University School of Medcine, Shanghai, 201801 China; 2 Department of General Surgery, Ruijin Hospital, Shanghai Jiaotong University School of Medicine, Shanghai, 200025 China

**Keywords:** Colorectal carcinoma, Lipopolysaccharide, miR-200c-3p, Exosome, ZEB-1

## Abstract

**Background:**

Colorectal cancer (CRC) is a leading cancer and a major cause of death. Lipopolysaccharide (LPS), an abundant component in gut microbiome, is involved in CRC progression and metastasis, potentially through regulating the miRNA composition of CRC-derived exosomes. In this study, we aimed to identify miRNA species in exosome which regulates CRC progression after LPS stimulation.

**Results:**

Firstly, we discovered a shift of miRNA profile in CRC exosome after LPS stimulation. Among the differentially expressed miRNAs, we identified miR-200c-3p as a potential key regulator of CRC progression and metastasis. Retrospective analysis revealed that miR-200c-3p was elevated in CRC tumor tissues, but decreased in the serum exosome in CRC patients. In vitro experiments demonstrated that exosomal miR-200c-3p expression did not influence CRC cell proliferation, but negatively regulated their capacity of migration and invasion in the presence of LPS. miR-200c-3p level in exosome influenced exosomal expression of *Zinc finger E-box-binding homeobox-1* (*ZEB-1)* mRNA, one of the miR-200c targets which affects migration and invasion capacity, and further altered ZEB-1 protein expression in CRC cell. In addition, exosomal miR-200c-3p promotes apoptosis of HCT-116 cells.

**Conclusions:**

Our findings indicate that exosomal miR-200c-3p inhibits CRC migration and invasion, and promotes their apoptosis after LPS stimulation. It is suggested as a potential diagnostic marker and therapeutic target of CRC.

## Background

Colorectal cancer (CRC) is the third most common cancer in the world and causes more than 600,000 deaths every year, particularly in elderly group [1, 2]. In physiological status, more than 100 trillion commensal microorganisms reside in the gastrointestinal tract and contribute to the maintenance of normal gut functions [3]. However, undesired alterations of the population, abundance and metabolic products in gut microbiome can significantly promote carcinogenesis and progression of CRC [4–7]. Lipopolysaccharide (LPS) is a key component of the outer membrane in Gram negative bacteria thus is widely distributed in gut microbiome [8]. Notably, it plays a crucial role in promoting CRC progression and metastasis through toll-like receptor 4 (TLR4)-mediated immune response [9–11].

Exosome is a nano-sized subtype of extracellular vesicles (EVs), which is released by almost all cell types including cancer cells [12, 13]. It contains various proteins and RNAs such as microRNA (miRNA) and long noncoding RNA (lncRNA) [12, 13]. In recent years, exosome has been identified as a major regulator of CRC progression [14]. Accumulating evidences have demonstrated that LPS can modulate exosome functions by altering exosomal composition [15, 16], particularly the miRNA profile [17, 18]. Therefore, it is of high interest to figure out whether LPS can regulate CRC progression and metastasis through specific miRNA species in CRC-secreted exosome.

In this study, we aimed to investigate the miRNA profile change in the exosomes from HCT-116 CRC cell after LPS stimulation. We identified miR-200c-3p as one of the key exosomal miRNAs in response to LPS. We also performed retrospective analysis and observed elevated miR-200c level in tumor tissue but decreased level in serum exosome from CRC patients. Our data suggested that miR-200c-3p can negatively regulate HCT-116 cell migration and invasion upon LPS stimulation in an exosome-dependent manner. This study will provide a novel insight on the miR-200c-3p function in CRC diagnosis and therapy.

## Results

### LPS did not change the morphorlogy of HCT-116 cell-derived exosome

**Fig. 1:**
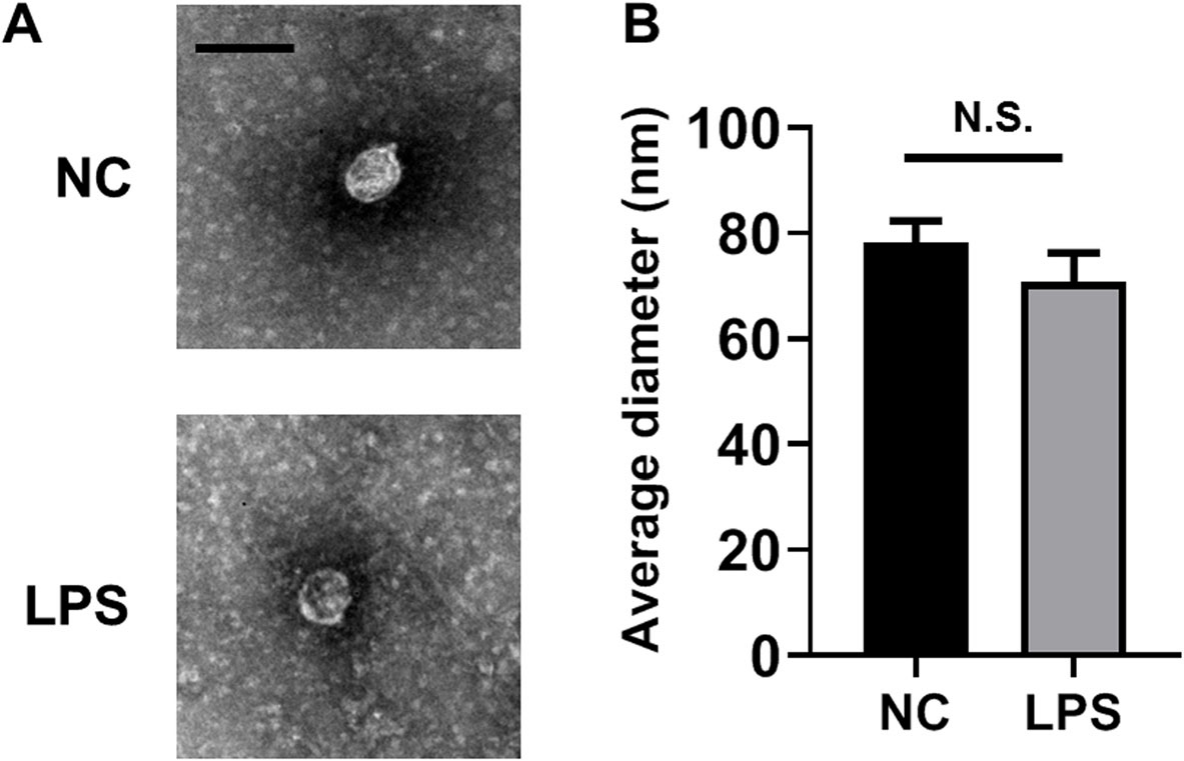
The morphology of HCT-116 cell-secreted exosome is not changed after LPS treatment. **a** Representative transmission electron microscopy (TEM) images of exosomes extracted from HCT-116 cells with or without LPS treatment. Scale bar: 100 nm; **b** Statistical analysis of exosome size between negative control (NC) and LPS groups (*n* = 20). Two-tailed t-test was performed. N.S., not significant

After 24 h LPS treatment, we extracted exosomes from HCT-116 cells and observed their morphology by transmission electron microscopy (TEM). There was no evident morphological abnormality of HCT-116 cell-derived exosome after LPS stimulation (Fig. 1a). In addition, no significant size difference was detected between control and LPS-treated groups (Fig. 1a and b).

### LPS induced the alteration of the miRNA profile in HCT-116 cell-derived exosome

**Fig. 2:**
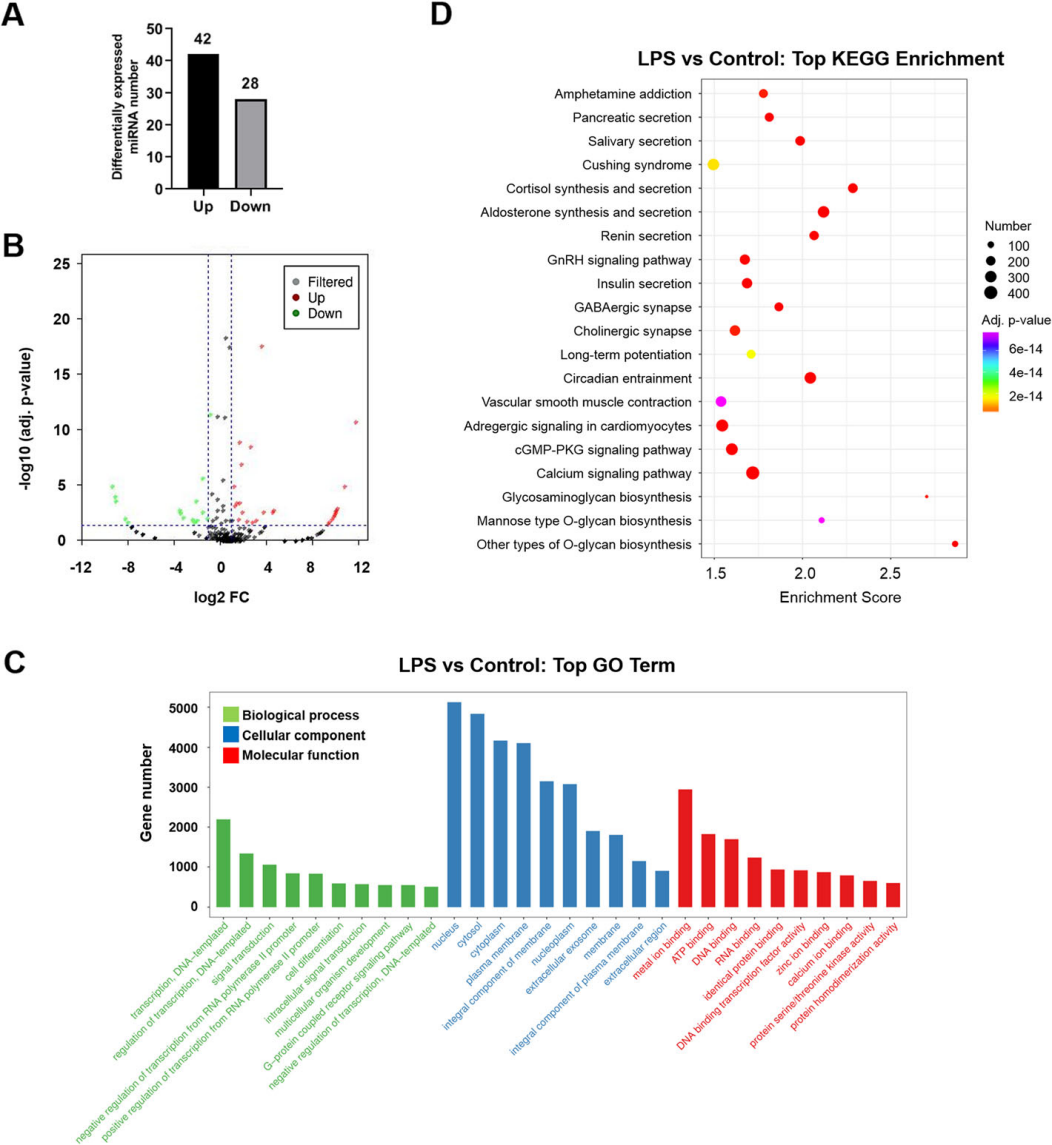
Bioinformatic analysis of miRNA transcriptome in the exosomes from HCT-116 cells with and without LPS treatment. **a** The total number of significantly up- and down regulated miRNA as presented in column graph. Differentially expressed miRNAs with |fold change (FC)| > 2 and adjusted *p*-value < 0.05 were considered as significant; **b.** Volcano plot of all up- and down regulated miRNAs; **c.** Gene set enrichment analysis (GSEA) of all differentially expressed miRNAs; **d.** KEGG enrichment analysis of all differentially expressed miRNAs

We further attempted to identify the miRNA profile change in the exosomes derived from LPS-treated cells. By RNA-seq analysis, more than 400 differentially expressed miRNAs in exosomes were discovered upon LPS stimulation (Additional file 1: Table S1). The miRNAs with fold change (FC) > 2 and adjusted *p*-value < 0.05 were selected as candidates (Additional file 1: Table S1). We eventually found 42 upregulated miRNAs and 28 downregulated miRNAs in HCT116 cell-derived exosome after LPS-treatment (Fig. 2a and b). To further predict their potential biological functions, all the differentially expressed miRNAs were subjected to gene set enrichment analysis (GSEA). GSEA results demonstrate that the selected miRNAs are involved in a number of cellular and molecular pathways (Fig. 2c). Additional KEGG enrichment analysis indicate that a number of metabolic pathways might be affected by these miRNAs (Fig. 2d).

### Differentially expressed miRNAs in gastro-intestinal cancer patients

**Fig. 3:**
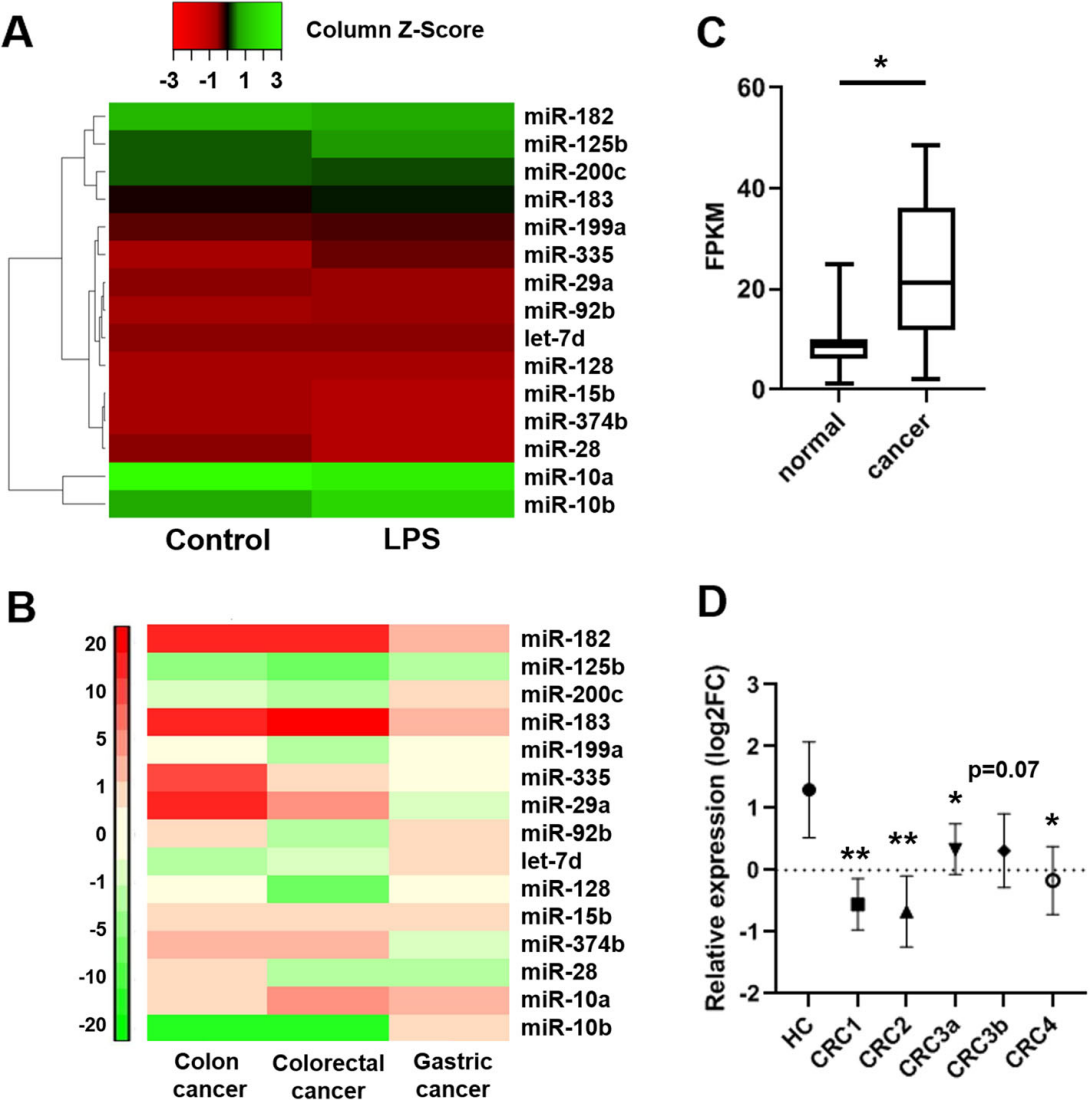
Selected miRNA expressions in gastro-intestinal cancer patients. **a** Heatmap of the selected top 15 differentially expressed miRNAs in control and LPS treated HCT-116-secreted exosomes; **b** Heatmap of selected top 15 differentially expressed miRNAs in colon cancer, gastric cancer and colorectal cancer. **c** miR-200c expression in CRC tissues and corresponding normal-appearing tissues from ten patients (*n* = 10). Data are acquired from GEO database (GSE126093). T-test was performed. * *p* < 0.05; **d** miR-200c expression in the serum exosome of CRC patients at stage 1 (*n* = 20), stage 2(*n* = 20), stage 3a(*n* = 20), stage 3b (*n* = 16) and stage 4 (*n* = 12). Healthy individuals (*n* = 11) were used as control (HC). FC: fold change. Data are acquired from GEO database (GSE39833). One-way ANOVA was performed. * *p* < 0.05; ** *p* < 0.01

We further selected 12 upregulated and 3 downregulated miRNAs from the 70 differentially regulated miRNAs according to their expression level in control group (transcript per million > 1), because lowly expressed miRNAs may have less significant biological contribution (Fig. 3a and Table 1). Uncharacterized novel miRNAs were also removed as their biological functions have not been identified. Analyzed by dbDEMC, a database of differentially expressed miRNAs in human cancers, the selected 15 miRNAs show distinct expression patterns in three major gastrointestinal cancers, colon cancer, gastric cancer and CRC (Fig. 3b). Among these miRNAs, we particularly concentrated on miR-200c-3p, because it has been suggested to play controversial roles in CRC [19–24]. Moreover, it may have potential diagnostic value to identify CRC stages [25–27]. Using public gene expression database, we noticed that miR-200c expression in CRC patient was significantly increased in tumor tissue (Fig. 3c). However, miR-200c level in serum exosome was strikingly lower in CRC patients comparing to healthy individuals (Fig. 3d). As miR-200c-5p level is below detection limit in miR-seq datasets, we only focused on miR-200c-3p for further analysis. In addition, we confirmed that exosomal miR-200c-3p level was elevated after LPS treatment not only in HCT-116 cells, but also in other CRC cell lines including HT-29 and SW480 (Additional file 2: Fig. S1), indicating a general response in CRC.

**Table 1:** Selected differentially expressed miRNAs in CRC-derived exosomes upon LPS induction

miRNA name	Log2(FC)	Adjusted ***p***-value
hsa-miR-335-5p	2.52234	2.76E-09
hsa-miR-10b-5p	1.92742	7.27E-26
hsa-miR-92b-3p	1.777154	0.002396
hsa-miR-183-5p	1.687399	1.14E-07
hsa-miR-125b-5p	1.595404	1.20E-09
hsa-miR-199a-5p	1.554762	0.000317
hsa-miR-128-3p	1.535396	0.060658
hsa-let-7d-3p	1.477946	0.011477
hsa-miR-200c-3p	1.288083	0.000317
hsa-miR-29a-3p	1.192192	0.191724
hsa-miR-182-5p	1.149881	0.000566
hsa-miR-10a-5p	1.07339	1.16E-05
hsa-miR-374b-5p	−1.20013	0.008328
hsa-miR-28-3p	−1.67406	2.09E-06
hsa-miR-15b-3p	−2.20013	0.000236

### Exosomal miR-200c-3p did not affect HCT-116 cell proliferation after LPS treatment

**Fig. 4:**
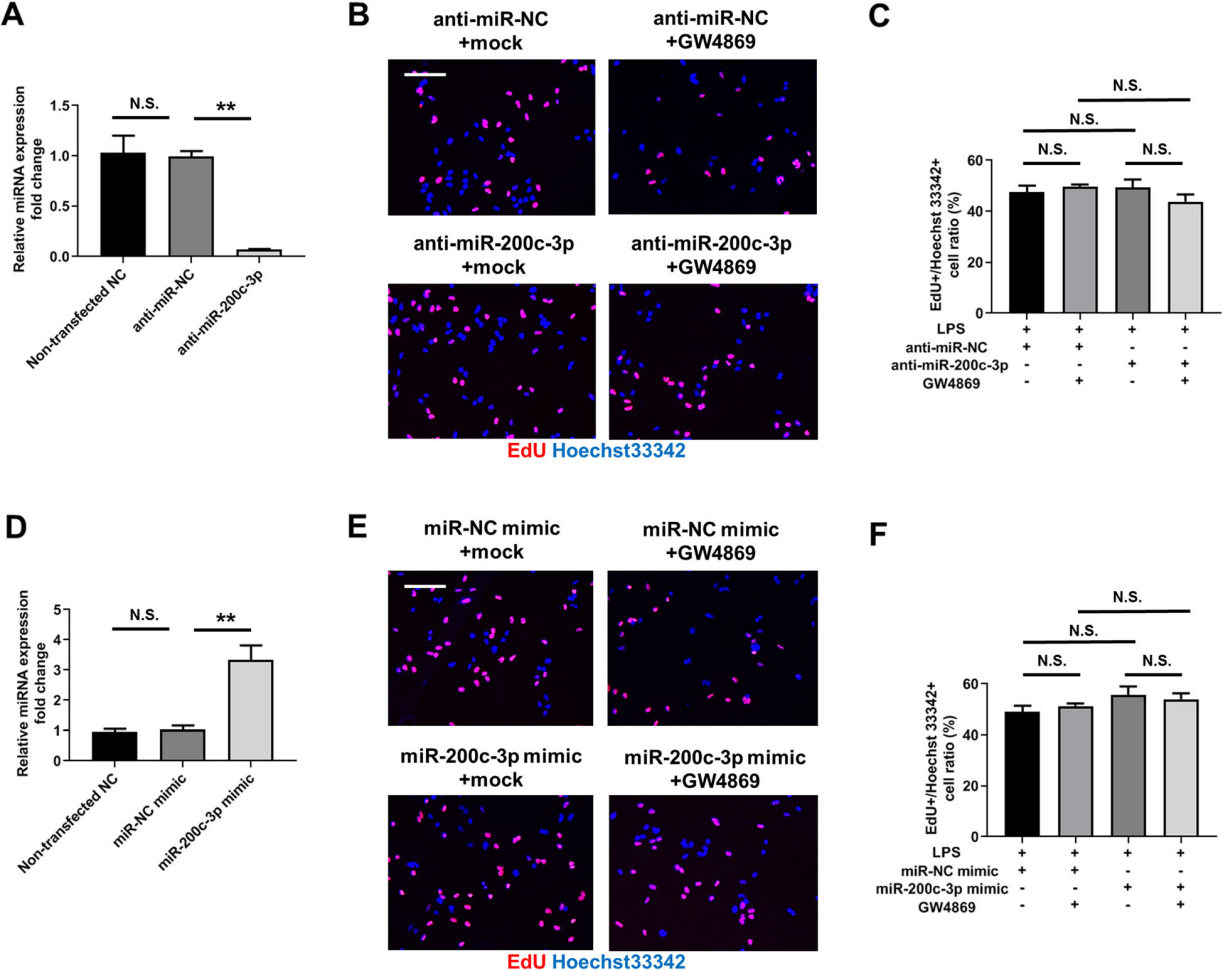
Exosomal miR-200c-3p expression did not change HCT-116 proliferation. Cells were treated with anti-miRNA or miRNA mimics for 24 h, and then treated with LPS and EdU, together with mock or exosome inhibitor GW4869 for additional 24 h as indicated in the Fig. **a**. Expression of miR-200c-3p in the exosomes of HCT-116 cells without transfection, or after transfection with anti-miR-NC (negative control) or anti-miR200c-3p (*n* = 3). N.S., not significant; ** *p* < 0.01; **b** Representative images of anti-miRNA-transfected HCT-116 cells labeled with EdU (red) and Hoechst 33342 (blue) 24 h after drug treatment. Scale bar: 100 μm; **c** Statistical analysis of EdU+/ Hoechst 33342+ cell ratio between groups with indicated treatment in **(B)** (*n* = 3). Two-way ANOVA was performed. N.S., not significant; **d** Expression of miR-200c-3p in the exosomes of HCT-116 cells without transfection, or after transfection with miR-NC (negative control) mimic or miR200c-3p mimic (*n* = 3). N.S., not significant; ** *p* < 0.01; **e** Representative images of miRNA mimic-transfected HCT-116 cells labeled with EdU (red) and Hoechst 33342 (blue) 24 h after drug treatment. Scale bar: 100 μm; **f** Statistical analysis of EdU+/ Hoechst 33342+ cell ratio between groups with indicated treatment in **(E)** (*n* = 3). Two-way ANOVA was performed. N.S., not significant

Next, we aimed to investigate the effects of exosomal miR-200c-3p on CRC proliferation, migration and invasion in the presence of LPS stimulation. We first attempted to downregulate exosomal miR-200c-3p expression by transfecting anti-miR-200c-3p into HCT-116 cells and by measuring miR-200c-3p level in extracted exosomes. As shown in Fig. 4a, anti-miRNA negative control (NC) did not influence miR-200c-3p expression in exosome when comparing to non-transfected sample, while anti-miR-200c-3p transfection remarkably reduced miR-200c-3p level (Fig. 4a). After miR-200c-3p knockdown, we exposed all the cells with LPS, and simultaneously blocked exosome secretion by GW4869, a standard exosome release inhibitor [28], to examine if miR-200c-3p function was dependent on exosome secretion. 5-ethynyl-2′-deoxyuridine (EdU) was incorporated into the cells to monitor cell proliferation. After 24 h drug treatment, no obvious difference was observed between all four groups (Fig. 4b and c), indicating a minor role of exosomal miR-200c-3p in the proliferation of CRC in current experimental conditions. We further overexpressed miR-200c-3p by transfecting miR-200c-3p mimic into HCT-116 cells, and elevated level of miR-200c-3p was detected in extracted exosomes (Fig. 4d). Similarly to knockdown experiment, neither miR-200c-3p overexpression nor GW4869 treatment altered the proliferation rate of HCT-116 cells (Fig. 4e and f).

### Exosomal miR-200c-3p inhibited HCT-116 cell migration after LPS treatment

**Fig. 5:**
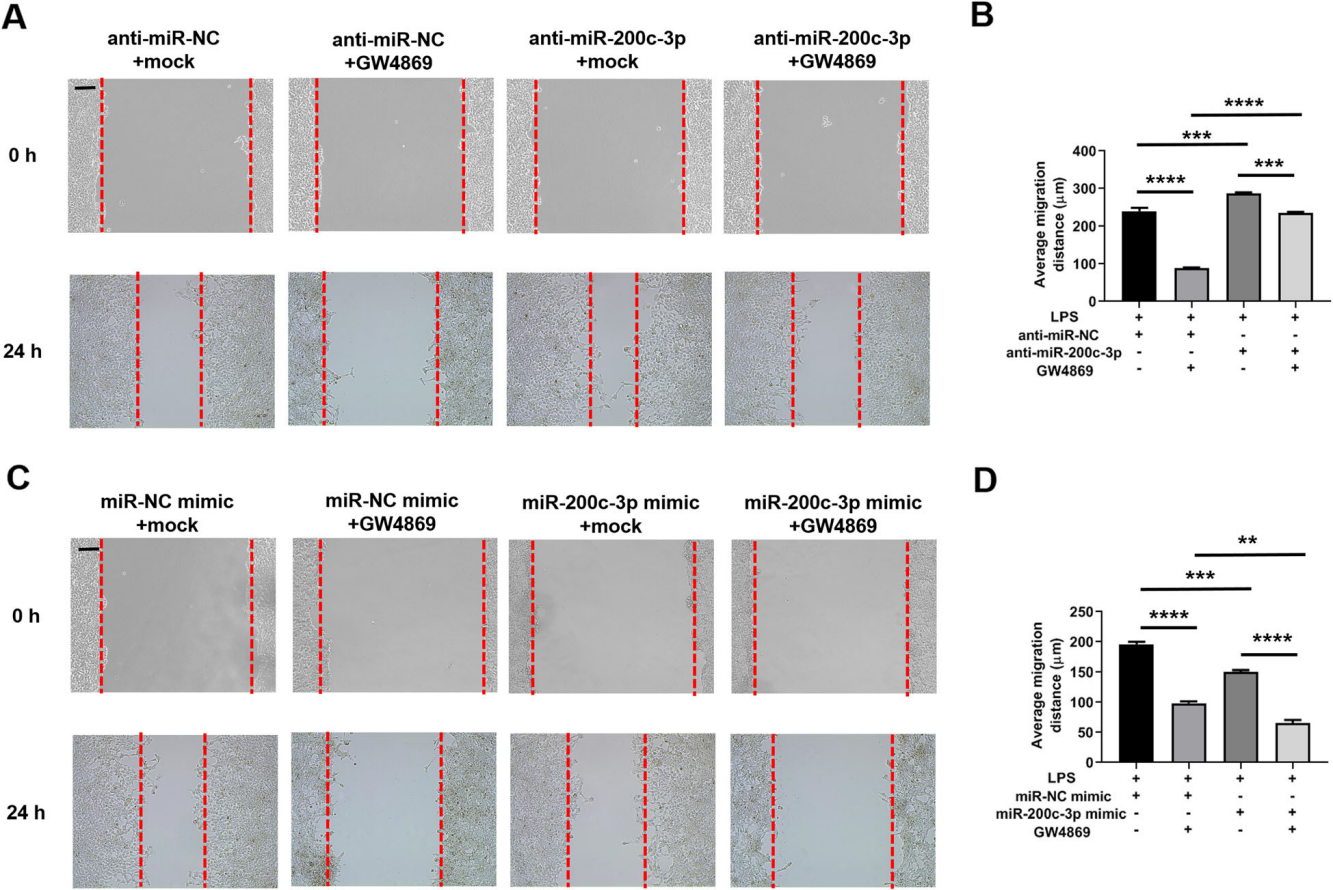
Exosomal miR-200c-3p inhibited HCT-116 migration. Cells were treated with control anti-miRNA or miRNA mimics for 24 h, induced a wound, and then treated with LPS together with mock or exosome inhibitor GW4869 for additional 24 h as indicated in the Fig. **a** Representative images of wound gap at 0 h and 24 h after drug treatment in anti-miRNA-transfected group. Scale bar: 100 μm; **b** Statistical analysis of wound gap distance (μm) between groups with indicated treatment in **(A)** (*n* = 3). Two-way ANOVA was performed. *** *p* < 0.0001; **** *p* < 0.0001; **c** Representative images of wound gap at 0 h and 24 h after drug treatment in miRNA mimic-transfected group. Scale bar: 100 μm; **d** Statistical analysis of wound gap distance (μm) between groups with indicated treatment in **(C)** (*n* = 3). Two-way ANOVA was performed. ** *p* < 0.01; *** *p* < 0.0001; **** *p* < 0.0001

Next, we checked whether exosomal miR-200c-3p affected CRC migration by wound healing assay. The same experimental group settings were applied as proliferation assay but without EdU incorporation. In miR-200c-3p knockdown experiment, cells significantly migrated after 24 h in control group (Fig. 5a and b). The inhibition of exosome secretion strikingly suppressed cell migration (Fig. 5a and b), consistent with previous reports [14]. However, downregulation of miR-200c-3p increased migration rate of HCT-116 cells, and considerably removed the effect of exosome inhibition (Fig. 5a and b). In contrast, miR-200c-3p mimic remarkably suppressed cell migration, and exosome secretion inhibition by GW4869 further strengthened this effect (Fig. 5c and d). In sum, miR-200c-3p negatively modulated the migration of CRC cells, and this inhibitory effect was executed particularly in the secreted exosome.

### Exosomal miR-200c-3p inhibited HCT-116 invastion after LPS treatment

**Fig. 6:**
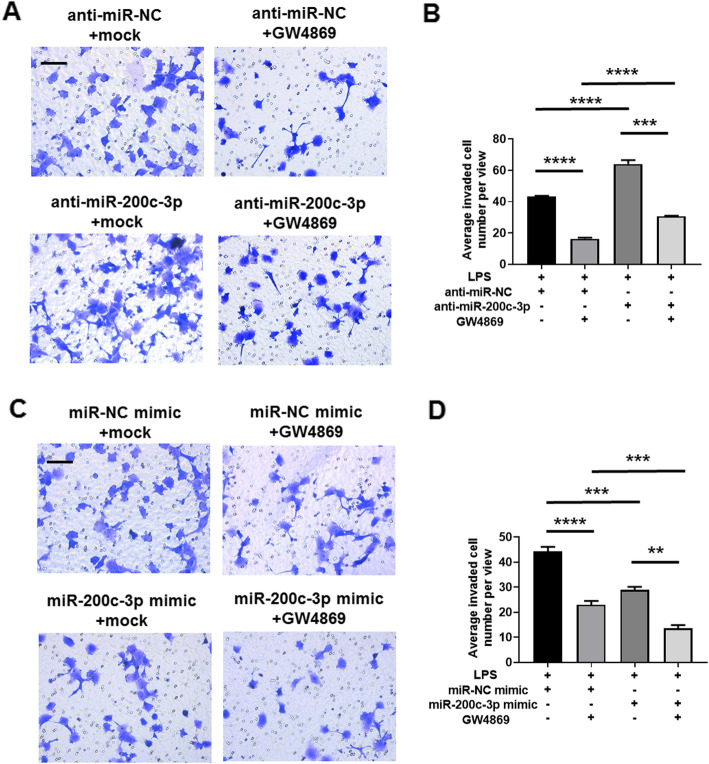
Exosomal miR-200c-3p inhibited HCT-116 invasion. Cells were treated with control anti-miRNA or miRNA mimics for 24 h, and then transferred to Matrigel-coated Transwell chamber, treated with LPS together with mock or exosome inhibitor GW4869 for additional 24 h as indicated in the Fig. **a** Representative images of stained invaded cells 24 h after drug treatment in anti-miRNA-transfected group. Scale bar: 50 μm; **b** Statistical analysis of invaded cell number between groups with indicated treatment in **(A)** (*n* = 3). Two-way ANOVA was performed. *** *p* < 0.001; **** *p* < 0.0001; **c** Representative images of stained invaded cells 24 h after drug treatment in miRNA mimic-transfected group. Scale bar: 50 μm; **d** Statistical analysis of invaded cell number between groups with indicated treatment in **(C)** (*n* = 3). Two-way ANOVA was performed. ** *p* < 0.01; *** *p* < 0.001; **** *p* < 0.0001

As exosomal miR-200c-3p showed inhibitory effects on migration capacity of HCT-116 cells, we speculated whether exosomal miR-200c-3p level also influenced CRC cell invasion. In Transwell invasion assay, more invaded cells were observed after miR-200c-3p knockdown (Fig. 6a and b). Similar to wound healing assay, the suppression of exosome secretion also resulted in a decrease of invasion ability, but this effect was alleviated by miR-200c-3p downregulation (Fig. 6a and b). On the contrary, overexpression of miR-200c-3p reduced invasion capacity both in the absence and presence of GW4869 treatment (Fig. 6c and d). Our data suggested that, similar to migration assay, miR-200c-3p decreased the invasion of HCT-116 cells, and this effect was also mediated by exosome.

### Exosomal miR-200c-3p inhibited ZEB-1 expression

**Fig. 7:**
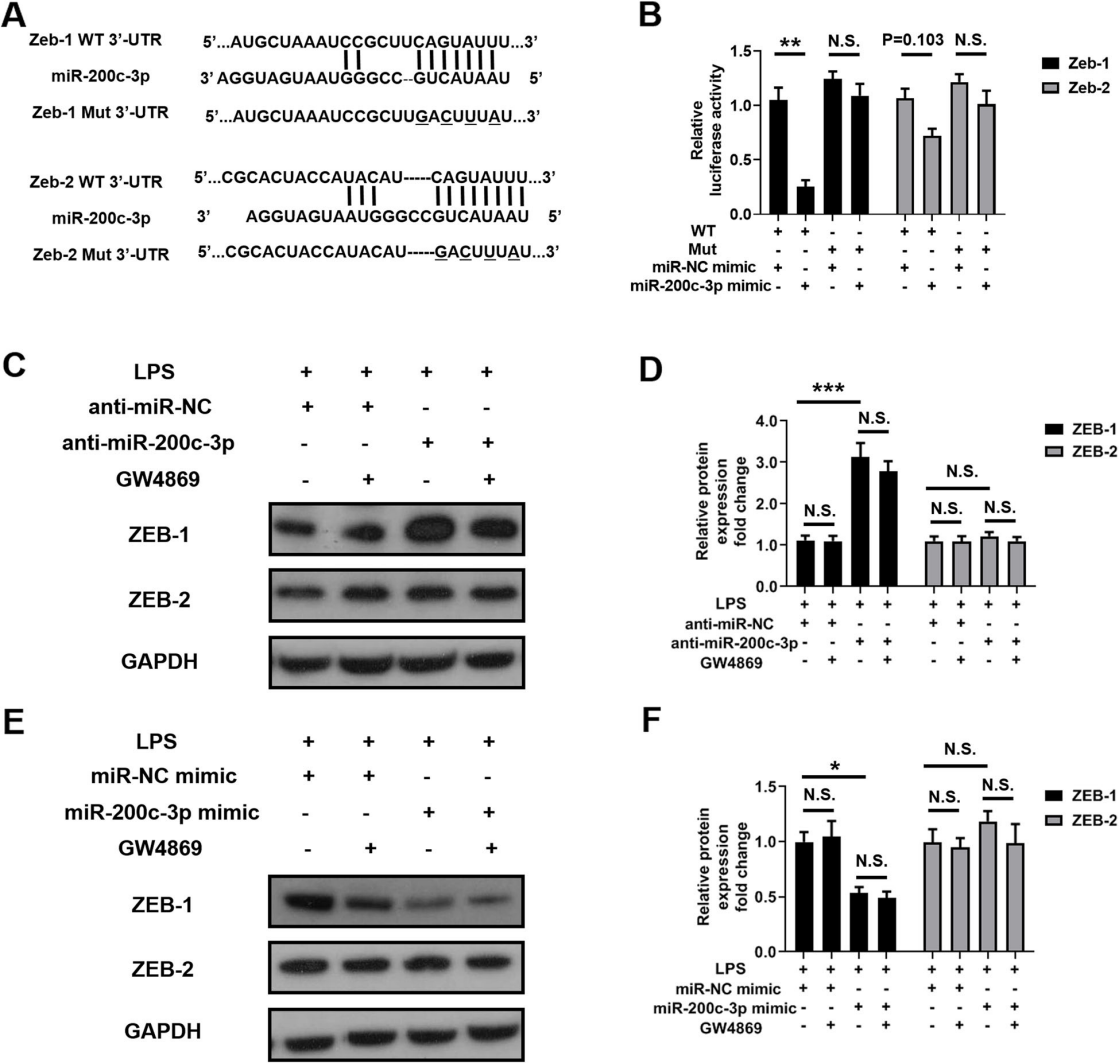
Exosomal miR-200c-3p inhibited ZEB-1 expression. **a** Predicted pairing of human miR-200c-3p to wildtype (WT) and mutated (Mut) *Zeb-1* 3’UTR and *Zeb-2* 3’UTR. **b** Dual luciferase gene reporter assay in HCT-116 cells co-transfected with indicated 3’UTR constructs and miRNA mimics (*n* = 3). Two-way ANOVA was performed for statistical analysis. N.S., not significant; ** *p* < 0.01. **c** Representative Western blot of ZEB-1 and ZEB-2 in anti-miRNA-transfected HCT-116 cell lysates with indicated treatments. GAPDH was used as loading control; **d** Statistical analysis of band intensities in **(C)** (*n* = 3). One-way ANOVA was performed. N.S., not significant; *** *p* < 0.001; **e** Representative Western blot of ZEB-1 and ZEB-2 in miRNA mimic-transfected HCT-116 cell lysates with indicated treatments. GAPDH was used as loading control; **f** Statistical analysis of band intensities in **(E)** (*n* = 3). One-way ANOVA was performed. N.S., not significant; * *p* < 0.05

Zinc finger E-box-binding homeobox 1 (ZEB-1) and Zinc finger E-box-binding homeobox 2 (ZEB-2) are well-known drivers of epithelial-mesenchymal transition (EMT) [29], which not only promote normal embryonic development but also contribute to cancer progression and metastasis [30–32]. Both ZEB1 and ZEB 2 can be regulated by miR-200 family, including miR-200c [33, 34]. A recent study has identified the presence of *ZEB-1* mRNA in cancer cell-derived exosomes [35]. Therefore, we hypothesized that miR-200c-3p level in exosome may also downregulate exosomal *Zeb-1* and/or *Zeb-2* mRNA levels, and further decrease their protein products in cytoplasm. The dual luciferase reporter assay showed that miR-200c-3p can target 3′-UTR of widetype *Zeb-1* mRNA, but not significantly with *Zeb-2* mRNA in HCT-116 cells (Fig. 7a and b). Mutated pairing region in 3′-UTR compeletely abolished the pairing between miR-200c-3p and 3′-UTR of both *Zeb-1* and *Zeb-2* mRNA (Fig. 7a and b). In cell lysate, ZEB-1 and ZEB-2 protein levels were not regulated by exosome inhibitor (Fig. 7c, d e and f, Additional files 3 and 4). Consistently, only ZEB-1, but not ZEB-2 protein expression was affected by miR-200c-3p level (Fig. 7c, d e and f, Additional file 3 and 4). Taken together, our data suggested that miR-200c-3p reduced *ZEB-1* mRNA level in exosome, and further resulted in decreased ZEB-1 protein expression. The reduced ZEB-1 level may contribute to impaired migration and invasion patterns of HCT-116 cells.

### Exosomal miR-200c-3p promotes HCT-116 apoptosis after LPS treatment

**Fig. 8:**
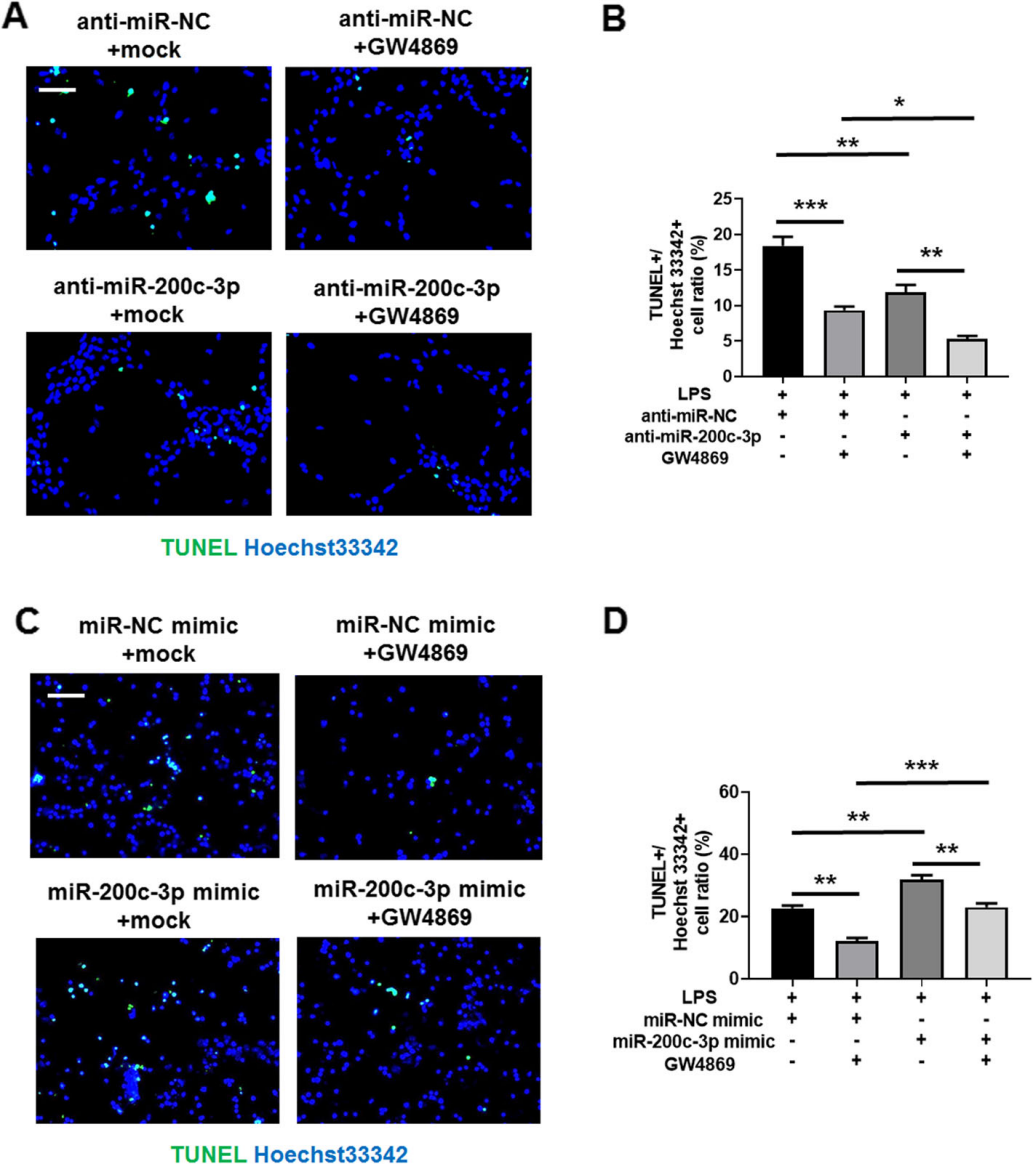
Exosomal miR-200c-3p promotes HCT-116 apoptosis. Cells were treated with control anti-miRNA or miRNA mimics for 24 h, and then treated with LPS together with mock or exosome inhibitor GW4869 for additional 24 h as indicated in the Fig. **a.** Representative images of HCT-116 cells labeled with TUNEL (green) and Hoechst 33342 (blue) 24 h after drug treatment in anti-miRNA-transfected group. Scale bar: 50 μm; **b.** Statistical analysis of TUNEL+/ Hoechst 33342 cell ratio between groups with indicated treatment in **(A)** (*n* = 3). Two-way ANOVA was performed. * *p* < 0.05; ** *p* < 0.01; *** *p* < 0.001; **c.** Representative images of HCT-116 cells labeled with TUNEL (green) and Hoechst 33342 (blue) 24 h after drug treatment in miRNA mimic-transfected group. Scale bar: 50 μm; **d.** Statistical analysis of TUNEL+/ Hoechst 33342 cell ratio between groups with indicated treatment in **(C)** (*n* = 3). Two-way ANOVA was performed. ** *p* < 0.01; *** *p* < 0.001

Finally, we checked whether exosomal miR-200c-3p can affect the apoptosis of HCT-116 cells. As LPS can induce apoptosis, we calculated TUNEL+/ Hoechst 33342 apoptotic cell ratio after LPS treatment. As shown in Fig. 8, less apoptotic cell ratio was observed after miR-200c-3p knockdown (Fig. 8a and b). The suppression of exosome secretion can also decrease apoptotic ratio, and this effect was strengthened by silencing miR-200c-3p (Fig. 8a and b). On the other hand, mimic miR-200c-3p expression significantly increased apoptotic cell ratio both in the absence and presence of GW4869 (Fig. 8c and d). Our data indicated that both cytoplasmic and exosomal miR-200c-3p can promote LPS-induced apoptosis in HCT-116 cells.

## Discussion

Epigenetic changes remarkably contribute to the development of CRC [36–38]. Colon contains around 70% of the host’s microorganisms [6], which can significantly alter epigenetic modifications in colon epithelial cells when their homeostasis is disrupted [7]. These alterations are able to drive the carcinogenesis, progression and metastasis of CRC [7]. Histone modification, DNA methylation and non-coding RNA including miRNA are the three main factors in epigenetic regulation [39]. MiRNA coordinates with the other two factors and triggers various physiological and pathological processes [39]. Recent studies have revealed a bidirectional regulatory mechanism between commensal gut microbiota and miRNA [40, 41], by which the physiological status of gut epithelial cells is changed, leading to detrimental immune responses and CRC carcinogenesis [42, 43]. When most investigatiors focus on the biochemical changes inside the cell, our data provides evidence of miRNA profiling change particularly in the exosomes of CRC after LPS stimulation (Fig. 2). Interestingly, the selected miRNAs in Table 1 may play contradict roles in the progression of CRC. For example, miR-10a-5p, miR-10b-5p and miR-125b-5p all belong to miR-10 family [44–46]. While miR-10b-5p normally promotes CRC invasion [44], miR-10a-5p and miR-125b-5p appear to suppress CRC invasion and metastasis [45, 46]. Notably, miR-10b-5p and miR-125b-5p are upregulated while miR-10a-5p is downregulated in CRC exosome after LPS stimulation (Table 1). Based on these results, even miRNA members from a single family may have opposite effects on CRC progression. Therefore, it is feasible to focus on a specific miRNA in a defined biological context before investigating the complexity of miRNA network. Furthermore, exosome is known to promote CRC migration and invasion in previous publication [14] and in our results (Fig. 5 and Fig. 6). Our new data will deepen our understanding on the epigenetic changes in LPS-induced CRC within a complex system, including cell-to-cell communications.

miR-200 family is upregulated in the circulation of various cancer patients, including ovarian, prostate, pancreatic, and colorectal cancers [47]. Recently, it has been found to regulate exosome-mediated metastasis in breast cancer [47]. Since only miR-200c, but not its counterpartners miR-200a and miR-200b, is on the screening list (Table 1), we searched literatures exclusively on miR-200c-mediated effects on CRC proliferation, invasion and metastasis. In the past decade, although quite a few studies have focused on the function of miR-200c in CRC, its role still remains ambiguous. The expression level of miR-200c varies in different CRC samples, and its functions in CRC cell proliferation, apoptosis, migration and invasion are also contradict in different experimental settings [19–24]. In addition, the role of miRNA in exosome may be distinct from that in cytosol, making it difficult to understand its biological functions without a specific context. Therefore, it is urgent to elucidate the exact role of miR-200c in CRC-released exosomes, which may serve as a biomarker and therapeutic target. In this study, we observed an upregulation of miR-200c expression in CRC exosome upon LPS stimulation (Additional file 2: Fig. S1). In clinical samples, miR-200c level in CRC patients is higher in tumor tissue but lower in serum exosome (Fig. 3c), indicating opposite roles of cellular and exosomal miR-200c. Further experiments revealed that the expression of miR-200c-3p in exosome resulted in impaired migration and invasion of CRC cell, although their proliferation capacity was not affected (Fig. 4, Fig. 5 and Fig. 6). Notably, the effects of miR-200c-3p can be masked when exosome secretion was suppressed (Fig. 5 and Fig. 6), suggesting a potential function of miR-200c-3p in exosome-mediated effect of CRC metastasis. The expression of miR-200c-3p in exosome potentially reduces the tumor-driving function of exosome, which is probably mediated by ZEB-1 protein (Fig. 7). Interestingly, as exosomal miR-200c-3p can be enhanced by LPS, it seems that the effects of LPS on CRC development are also complex, with both beneficial and unfavorable aspects. Considering LPS serves as a natural apoptosis inducer, cytoplasmic and exosomal miR-200c-3p can both stimulate CRC apoptosis (Fig. 8) in combination with the inhibitory effects on migration and invasion (Fig. 5 and Fig. 6). Our results suggests miR-200c-3p as an anti-cancer target at various biological levels. Since miR-200c-5p expression level is low in CRC-derived exosomes, it was not investigated in this study. However, it may also play either synergistic or antagonistic role with miR-200c-3p in exosomes from other cancer types.

## Conclusions

In general, our study evokes the considerations of miR-200c-3p in exosome instead of in cytosol, in the context of CRC development with LPS-stimulation. Our data support the idea that, exosomal miR-200-3c prevents CRC migration and metastasis, as well as its survival in the presence of LPS stimulation. Our knowledge on exosomal miR-200c-3p provides a novel avenue in the field of CRC therapy for both basic researchers and clinicians.

## Methods

### Cell culture and drug treatment

Human colorectal carcinoma cell line HCT-116 was purchased from Sigma-Aldrich (Catalogue No. 91091005). Human colorectal carcinoma cell lines HT-29 and SW480 were purchased from Beyotime (Catalogue No. C6410 and C6915, respectively). HCT-116 cells were cultured in McCoy’s 5A medium (KeyGEN BioTECH, KGM4892–500) supplemented with 2 mM L-glutamine (KeyGEN BioTECH, KGY0042) and 10% fetal bovine serum (Excell Bio, FCS500) at 37 °C with 5% CO2. HT-29 and SW480 cells were cultured in RPMI-1640 medium supplemented with 2 mM L-glutamine and 10% fetal bovine serum (Sigma-Aldrich, SLM-240-B) at 37 °C with 5% CO2. When indicated, cells were treated with 10 ng/mL lipopolysaccharides (LPS) (KeyGEN BioTECH, KGR0048) and exosome inhibitor GW4869 (MedChemExpress, HY-19363) for 24 h before harvesting.

### Anti-miRNA or mimic miRNA transfection

Negative control (NC) anti-miRNA or mimic miRNA, and miR200c-3p specific anti-miRNA or mimic miRNA were synthesized by GenePharma with the following sequences:

anti-miR-NC: CAGUACUUUUGUGUAGUACAA.

anti-miR-200c-3p: UCCAUCAUUACCCGGCAGUAUUA.

miR-NC mimic: UACAGCCUUAUACCAUGAAUGC.

miR-200c-3p mimic: UAAUACUGCCGGGUAAUGAUGGA.

Transfection of HCT-116 cells with anti-miRNAs or mimic miRNAs were performed with RNAi-Mate transfection reagent (GenePharma, G04001) for 24 h before LPS and GW4869 drug treatments.

### Exosome isolation

HCT-116 cells were briefly spin down, and large particles in supernatant were excluded with 0.8 μm Millex-AA syringe filter (Millipore, SLAA033SB). Exosomes were isolated with exoEasy Maxi Kit (Qiagen, 76,064) following the instructions of the manufacturer.

### Transmission electron microscopy (TEM)

Exosome negative staining was performed according to published method [48]. JEM1011 transmission electron microscope (JEOL) was used for imaging. Twenty exosome particles per group were chosen randomly from control and LPS groups, and their diameters were measured by Image J software (National Institutes of Health, NIH) for statistical analysis.

### miRNA sequencing and bioinformatic analysis

Total RNA including miRNA from exosome was purified with exoRNeasy Serum/Plasma Starter Kit (Qiagen, 77,023). miRNA library was constructed with QIAseq miRNA Library Kit (Qiagen, 331,502). miRNA-seq was performed on HiSeq X Ten (Illumina) with 150 paired-end (PE). Raw data were process to remove adaptor sequences and exclude reads < 21 bp or > 25 bp. Clean reads were aligned to miRBase (www.mirbase.org/). Differentially expressed miRNAs were filtered with the threshold fold change (FC) > 2 and adjusted *p*-value < 0.05. Column graph and volcano plot was prepared by GraphPad Prism 8. A further selection was performed with a cutoff of expression (> 1 transcript per million) in control group and a removal of uncharacterized novel miRNAs. Heatmap was generated via http://www.heatmapper.ca/expression. Gene set enrichment analysis (GSEA) was performed via http://www.webgestalt.org. KEGG enrichment analysis was performed via https://www.genome.jp/kegg/. The miRNA meta-profiling heatmap was generated by dbDEMC 2.0 database [49] via http://www.picb.ac.cn/dbDEMC/profiling.html. Retrospective analysis of miR-200c expression in CRC patients was performed using the metadata from GEO public database (https://www.ncbi.nlm.nih.gov/geo/). Datasets GSE126093 and GSE39833 were used.

### Quantitative PCR

Total RNA including mRNA and miRNA from exosome was isolated as described above. cDNA of mRNA was synthesized with HiScript II 1st Strand cDNA Synthesis Kit (Vazyme, R211). cDNA of miRNA was synthesized with miRNA 1st Strand cDNA Synthesis Kit (by stem-loop) (Vazyme, MR101–01). Quantitative PCR was performed with ChamQ Universal SYBR qPCR Master Mix (Vazyme, Q711–02) in 20 μL volume. Thermal cycles were run on 7500 Real-Time PCR System (Applied Biosystems) with the program: 95 °C 5 min for pre-denaturation; 95 °C 15 s, 60 °C 20 s, 72 °C 40 s for 40 cycles; followed by melting curve analysis. The primer sequences used for quantitative PCR were listed below:

*U6*-F: CTCGCTTCGGCAGCACA.

*U6*-R: AACGCTTCACGAATTTGCGT.

*miR-200c-3p*-F: AACAAGTAATACTGCCGGGTAATGA.

*miR-200c-3p*-R: CAGTGCAGGGTCCGAGGT.

According to previous publications [50], *U6* was used as an internal control of exosomal miRNA.

### Western blot

HCT-116 cells were harvested and washed with cold PBS. Cells were lysed in lysis buffer (1% Triton X-100, 50 mM Tris-HCl, 150 mM NaCl, protease inhibitor cocktail (Beyotime, P1006), pH 8.0) and total protein concentrations were determined by BCA Protein Assay Kit (Beyotime, P0011). Fifty μg total protein was separated by sodium dodecyl sulfate-polyacrylamide gel electrophoresis (SDS-PAGE) and transferred onto polyvinylidene difluoride (PVDF) membranes. The membranes were incubated with primary antibodies overnight at 4 °C, washed three times with PBST (PBS plus 0.1% Triton X-100) and then incubated with horseradish peroxidase (HRP)-conjugated secondary antibodies for 2 h at room temperature. After incubation, the membranes were washed three times with PBST and developed with enhanced chemiluminescence (ECL) substrate (Beyotime, P0018). The primary antibodies were listed below:

ZEB-1 antibody: Santa Cruz (sc-515,797).

ZEB-2 antibody: Santa Cruz (sc-271,984).

GAPDH antibody: Beyotime (AG019).

GAPDH was used as loading control.

### Proliferation assay

The proliferation of HCT-116 cells was measured with BeyoClick™ EdU Cell Proliferation Kit with Alexa Fluor 647 (Beyotime, C0081L). Briefly, 2 mL cells at the density of 1.5 × 10^5^ / mL were seeded in one well of 6-well plate with glass bottom and cultured overnight. After 24 h transfection with anti-miRNA or mimic miRNA, cells were treated with indicated drugs and 10 μM EdU for 24 h. Cells were then fixed with 4% paraformaldehyde (PFA) for 15 min, washed three times with PBS and permeabilized with 0.3% Triton X-100 in PBS for 15 min. EdU was detected with Click Additive Solution from the kit, and all the nuclei were counterstained with Hoechst 33342. Three random fields of each sample were acquired with 20x objective lens using fluorescent microscope (Zeiss, Axio Imager A1). The mean value of EdU+ / Hoechst 33342+ cell ratio from three fields was calculated for each experiment. Three independent experiments were performed.

### Wound healing assay

Two mL cells at the density of 1.5 × 10^5^ / mL were seeded in one well of 6-well plate and cultured overnight before anti-miRNA or mimic miRNA transfection. After transfection, cells were maintained in 10% fetal bovine serum for 24 h until the confluency reached 80–90%, and then switched to culture medium containing 1% fetal bovine serum to inhibit proliferation. A ventricle wound through cell layer was made by 200 μl pipette tip. Drugs were then added into the medium and cells were cultured for additional 24 h. The wound healing images with three random fields were acquired 0 h and 24 h after the addition of drugs using bright-field inverted microscope (Zeiss, Axio Vert.A1). Migration distance (μm) was calculated by substracting the gap distance at 24 h from that at 0 h. The mean value of migration distance (μm) from three fields was calculated for each experiment. Three independent experiments were performed.

### Transwell invasion assay

Invasion assay was performed with Matrigel pre-coated invasion chamber suited for 6-well plate (Corning, 354,481). Cells were cultured in 6-well plate at the density of 1.5 × 10^5^ / mL and transfected with anti-miRNA or mimic miRNA for 24 h. Then the cells were trypsinized and seeded into the Matrigel-coated chamber at the density of 5.0 × 10^5^ / mL and incubated with indicated drugs for 24 h. Non-invaded cells were scraped off with cotton swab, while invaded cells were fixed with 4% paraformaldehyde and stained with crystal violet. Three random fields were acquired with bright-field inverted microscope (Zeiss, Axio Vert.A1). The mean counted cell number from three fields was calculated for each experiment. Three independent experiments were performed.

### Dual luciferase reporter assay

The 3′-UTR regions of human *Zeb-1* and *Zeb-2* genes were cloned into psiCHECK2 plasmid (Promega, C8021) for luciferase reporter assay. The sequences of cloning primers were reported in previous publications [51, 52]. Multiple point mutations were obtained by QuickMutation Kit (Beyotime, D0206). The mutant sequences of *Zeb-1* and *Zeb-2* are shown in Fig. 7a. Constructed plasmids were co-transfected with miR-NC-mimic or miR-200c-3p mimic into HCT-116 cells for 24 h. The luciferase activity was measured by Dual Luciferase Reporter Gene Detection Kit (Beyotime, RG027). The signals from *Renilla* luciferase were normalized to the signals from firefly luciferase.

### Apoptosis assay

The apoptosis of HCT-116 cells was measured with One Step TUNEL Apoptosis Assay Kit (Beyotime, C1086). 2 mL cells at the density of 1.5 × 10^5^ / mL were seeded in one well of 6-well plate with glass bottom and cultured overnight. After 24 h transfection with anti-miRNA or mimic miRNA, cells were treated with LPS (also for the induction of apoptosis) and GW4869. After additional 24 h, cells were then subjected to the procedures of One Step TUNEL Apoptosis Assay Kit, and all the nuclei were counterstained with Hoechst 33342. Three random fields of each sample were acquired with 20x objective lens using fluorescent microscope (Zeiss, Axio Imager A1). The mean value of TUNEL+ / Hoechst 33342+ cell ratio from three fields was calculated for each experiment. Three independent experiments were performed.

### Statistical analysis

All experiments were performed in at least triplicates and presented as standard mean error (SEM). Two-tailed t-test with Welch’s correction was used for two group comparison with a single factor. One-way ANOVA followed by Tukey’s test was used for group comparison with a single factor but more than three groups. Two-way ANOVA followed by Tukey’s test was used for the group comparison with two factors. GraphPad Prism 8 was used for statistical analysis. *p*-value < 0.05 was considered as significant.

## Supplementary information

**Additional file 1: Table S1.** Full list of differentially expressed exosomal miRNAs after LPS stimulation in HCT-116 cells.

**Additional file 2: Figure S1.** Exosomal miR-200c-3p expression in HCT-116, HT-29 and SW480 CRC cell lines after LPS stimulation. Cells were treated with 10 ng/mL LPS for 24 h before isolating exosomes. Total RNAs were extracted from exosomes, and cDNAs were synthesized from miRNAs. Expression levels of miR-200c-3p in exosomes were measured by quantitative real-time PCR (*n* = 3). Two-tailed t-test was performed for statistical analysis. * *p* < 0.05.

**Additional file 3.** Uncropped Western blot in Fig. 7**c.** ZEB-1, ZEB-2 and GAPDH expressions in HCT-116 cells after indicated treatment.

**Additional file 4.** Uncropped Western blot in Fig. 7**e.** ZEB-1, ZEB-2 and GAPDH expressions in HCT-116 cells after indicated treatment.

## Data Availability

All data generated or analyzed in this study are included in this manuscript and supplementary information. The raw datasets are available from the corresponding author on reasonable request.

## Abbreviations

CRC: Colorectal cancer
ECL: Enhanced chemiluminescence
EdU: 5-Ethynyl-2′-deoxyuridine
EMT: Epithelial-mesenchymal transition
EV: Extracellular vesicles
FC: Fold change
GSEA: Gene set enrichment analysis
HRP: Horseradish peroxidase
lncRNA: Long noncoding RNA
LPS: Lipopolysaccharide
miRNA: microRNA
NC: Negative control
PE: Paired-end
PFA: Paraformaldehyde
PVDF: Polyvinylidene difluoride
SDS-PAGE: Sodium dodecyl sulfate-polyacrylamide gel electrophoresis
SEM: Standard mean error
TEM: Transmission electron microscopy
TLR4: Toll-like receptor 4
UTR: Untranslated region
ZEB-1: Zinc finger E-box-binding homeobox-1
ZEB-2: Zinc finger E-box-binding homeobox-2
