# Supplementary material for: Exosomal miR-200c-3p negatively regulates the migraion and invasion of lipopolysaccharide (LPS)-stimulated colorectal cancer (CRC)
Source: BMC Mol Cell Biol. 2020 Jun 29;21:48. doi: 10.1186/s12860-020-00291-0 (PMC7325272; doi:10.1186/s12860-020-00291-0)

**Additional file 2: Fig. S1.** **Exosomal miR-200c-3p expression in HCT-116, HT-29 and SW480 CRC cell lines after LPS stimulation.** Cells were treated with 10 ng/mL LPS for 24 h before isolating exosomes. Total RNAs were extracted from exosomes, and cDNAs were synthesized from miRNAs. Expression levels of miR-200c-3p in exosomes were measured by quantitative real-time PCR (n=3). Two-tailed t-test was performed for statistical analysis.

* p<0.05.


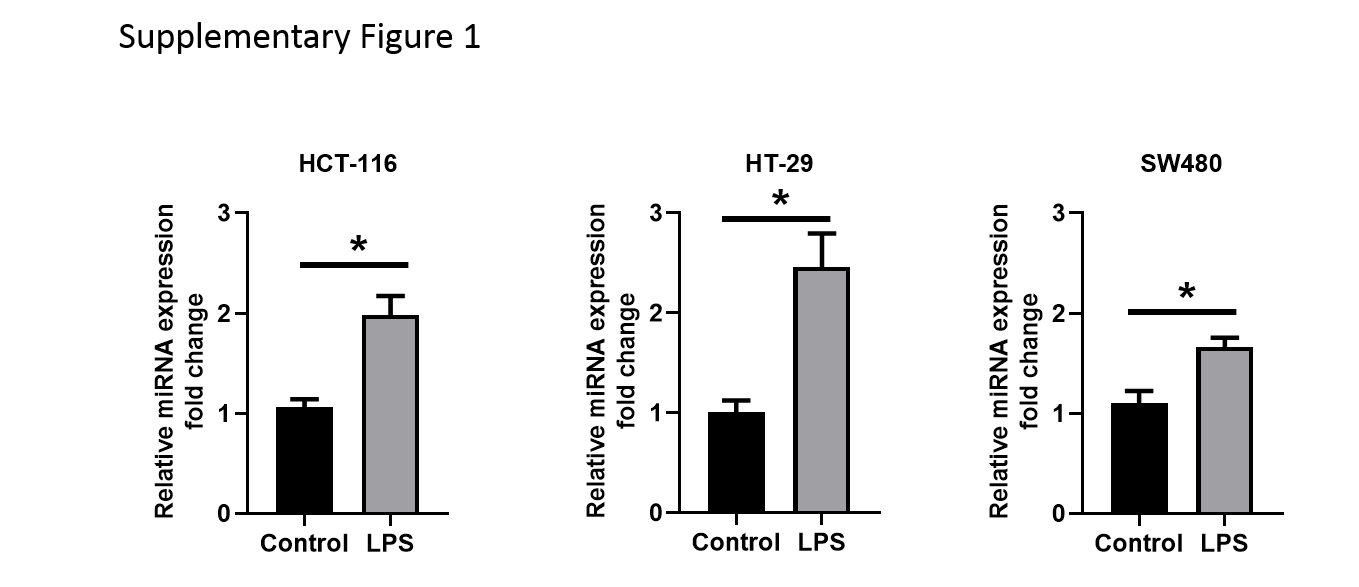

Supplement: Supplementary file 2 — Additional file 2: Figure S1. Exosomal miR-200c-3p expression in HCT-116, HT-29 and SW480 CRC cell lines after LPS stimulation. Cells were treated with 10 ng/mL LPS for 24 h before isolating exosomes. Total RNAs were extracted from exosomes, and cDNAs were synthesized from miRNAs. Expression levels of miR-200c-3p in exosomes were measured by quantitative real-time PCR (n = 3). Two-tailed t-test was performed for statistical analysis. * p < 0.05. [file 12860_2020_291_MOESM2_ESM.docx]
