# Supplementary figures and images for: Exosomal miR-200c-3p negatively regulates the migraion and invasion of lipopolysaccharide (LPS)-stimulated colorectal cancer (CRC)
Source: BMC Mol Cell Biol. 2020 Jun 29;21:48. doi: 10.1186/s12860-020-00291-0 (PMC7325272; doi:10.1186/s12860-020-00291-0)

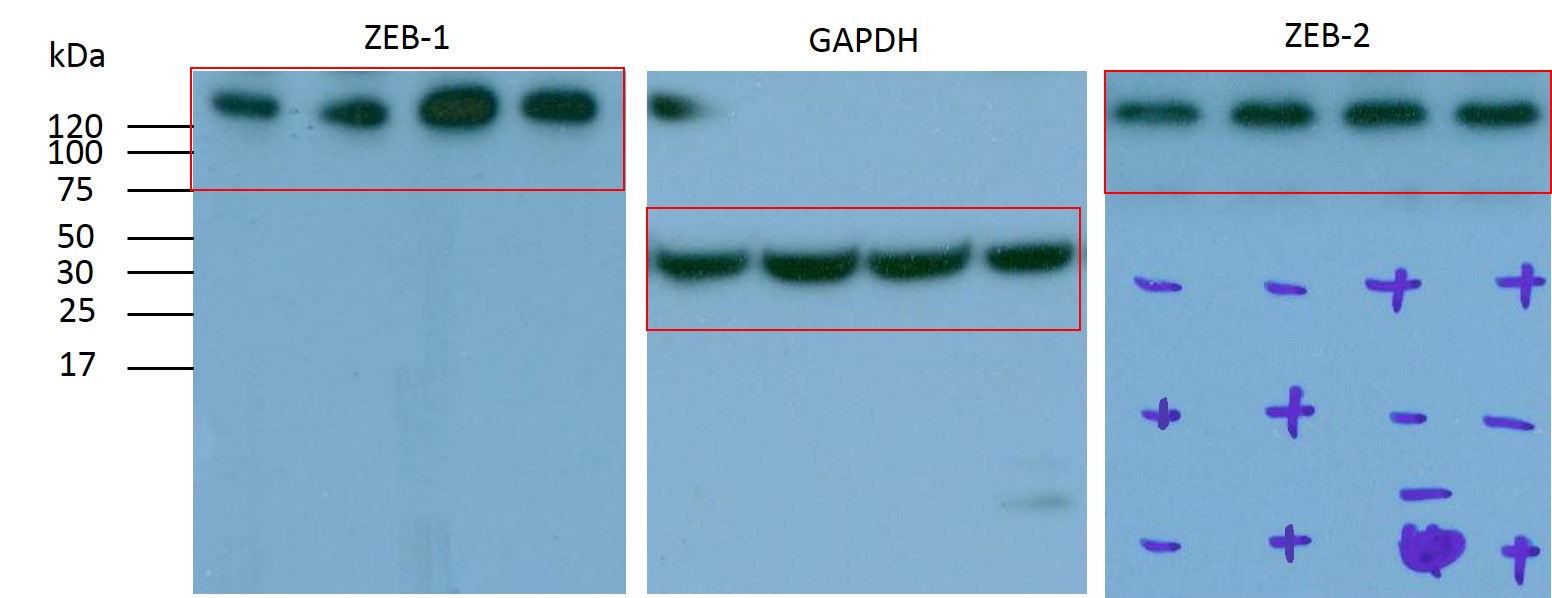

Supplement: Supplementary file 3 — Additional file 3. Uncropped Western blot in Fig. 7c. ZEB-1, ZEB-2 and GAPDH expressions in HCT-116 cells after indicated treatment. [file 12860_2020_291_MOESM3_ESM.jpg]

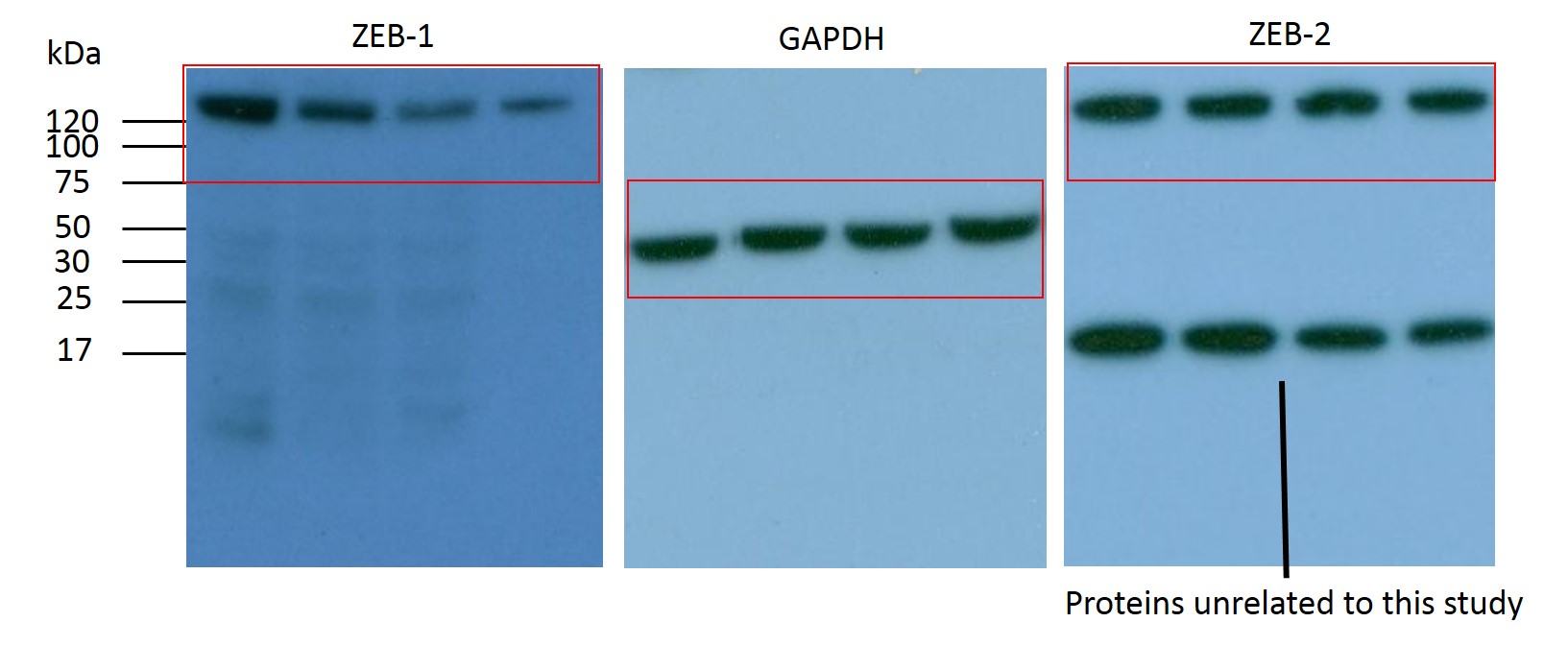

Supplement: Supplementary file 4 — Additional file 4. Uncropped Western blot in Fig. 7e. ZEB-1, ZEB-2 and GAPDH expressions in HCT-116 cells after indicated treatment. [file 12860_2020_291_MOESM4_ESM.jpg]
